# Conceivable difference in the anti-inflammatory mechanisms of lipocortins 1 and 5

**DOI:** 10.1155/S0962935193000158

**Published:** 1993

**Authors:** C. Becherucci, M. Perretti, E. Solito, C. L. Galeotti, L. Parente

**Affiliations:** 1 Department of Molecular Biology Immunobiological Research Institute Siena Siena 53100 Italy; 2 Department of Pharmacology Immunobiological Research Institute Siena Siena 53100 Italy; 3 Department of Biochemical Pharmacology The William Harvey Research Institute St. Bartholomew's Hospital Medical School Charterhouse Square London EC1M 6BQ UK

## Abstract

Human recombinant lipocortins (LCT) 1 and 5 have been expressed in a yeast secretion vector and purified by ion exchange chromatography. The action of the proteins has been investigated in two models of experimental acute inflammation in the rat: carrageenin induced paw oedema and zymosan induced pleurisy. The effects of the proteins on PGE_2_ release *in vitro* by rat macrophages stimulated with zymosan and on rat neutrophil chemotaxis induced by FMLP have also been assessed. LCT-1 significantly inhibited both paw swelling in carrageenin oedema and leukocyte migration in zymosan pleurisy. Moreover it showed a dose dependent, inhibitory effect on PGE_2_ release. Neutrophil chemotaxis was only weakly affected by LCT-1. Conversely LCT-5 did not reduce carrageenin oedema and slightly inhibited PGE_2_ release, but showed profound, dose dependent inhibitory activity on leukocyte migration in zymosan pleurisy and on neutrophil chemotaxis. These data suggest that LCT-1 acts mainly by interfering with arachidonic acid metabolism via the inhibition of phospholipase A_2_. The anti-inflammatory activity of LCT-5, at variance with LCT-1, may be due to a direct effect on cell motility in addition to the interference with arachidonic acid metabolism.


Mediators of Inflammation, 2, 109–113 (1993)

### Conceivable difference in the anti-inflammatory mechanisms of lipocortins 1 and 5

C. Becherucci, M. Perretti,* E. Solito, C. L. Galeotti,[1] and L. Parente[CA]

Departments of Pharmacology and [1]Molecular Biology, Immunobiological Research Institute Siena, 53100 Siena, Italy

**Present Address*: Department of Biochemical Pharmacology, The William Harvey Research Institute, St. Bartholomew's Hospital Medical School, Charterhouse Square, London EC1M 6BQ, UK

[CA] Corresponding Author

**HUMAN recombinant lipocortins (LCT) 1 and 5 have been expressed in a yeast secretion vector and purified by ion exchange chromatography. The action of the proteins has been investigated in two models of experimental acute inflammation in the rat: carrageenin induced paw oedema and zymosan induced pleurisy. The effects of the proteins on $PGE_2$ release *in vitro* by rat macrophages stimulated with zymosan and on rat neutrophil chemotaxis induced by FMLP have also been assessed. LCT-1 significantly inhibited both paw swelling in carrageenin oedema and leukocyte migration in zymosan pleurisy. Moreover it showed a dose dependent, inhibitory effect on $PGE_2$ release. Neutrophil chemotaxis was only weakly affected by LCT-1. Conversely LCT-5 did not reduce carrageenin oedema and slightly inhibited $PGE_2$ release, but showed profound, dose dependent inhibitory activity on leukocyte migration in zymosan pleurisy and on neutrophil chemotaxis. These data suggest that LCT-1 acts mainly by interfering with arachidonic acid metabolism via the inhibition of phospholipase $A_2$. The anti-inflammatory activity of LCT-5, at variance with LCT-1, may be due to a direct effect on cell motility in addition to the interference with arachidonic acid metabolism.**

**Key words:** Anti-inflammatory effect, Glucocorticoids, Lipocortin/annexin

### Introduction

Glucocorticoids are drugs endowed with a potent anti-inflammatory effect, although the mechanism of action is not completely understood. It has been proposed that they inhibit phospholipase $A_2$ ($PLA_2$) activity and thus prevent eicosanoid formation. The inhibitory effect of glucocorticoids on $PLA_2$ appears to be mediated by inducible inhibitory proteins called lipocortins (LCTs).[1] After the cloning and identification of the gene of lipocortin 1 (LCT-1) it has been shown that they belong to a larger family of proteins (annexins) with calcium and phospholipid binding properties, whose precise biological role is not yet clear.[2] *In vivo* anti-inflammatory activity has been reported in experimental inflammation for recombinant LCT-1[3] and for a purified 36 kDa protein immunologically related to lipocortin 5 (LCT-5).[4] Recently, by aligning the sequences of LCT-5 and uteroglobin (another anti-$PLA_2$ protein) a nine aminoacid conservative region has been identified. The peptide deriving from this region of LCT-5 (amino acids 204–212) has shown anti-inflammatory activity both *in vitro* and *in vivo* suggesting that it could represent the active site of the anti-inflammatory effect of LCT-5.[5] In this paper the anti-inflammatory mechanism of LCT-1 and LCT-5 has been investigated both *in vitro* and *in vivo*. The results confirm that the two proteins possess anti-inflammatory activity and suggest that they act differently in the control of experimental inflammation.

### Materials and Methods

*Expression and purification of the proteins:* Human LCT-1 and LCT-5 have been obtained as recombinant proteins by using a yeast expression/secretion system. The published cDNAs[6,7] for both proteins have been subcloned into the *Saccharomyces cerevisiae* secretion vector YEpsec 1 previously described.[8] Yeast cells transformed with the recombinant plasmids secrete the heterologous proteins into the culture medium containing galactose as inducer of expression. The proteins secreted in the yeast culture medium were purified by ion exchange chromatography. The medium containing LCT-1 was first applied to a pre-equilibrated 20 × 2.5 cm column of DEAE cellulose (DE-52 Whatman, Maidstone, UK) in 25 mM Tris buffer pH 7.7 and thoroughly washed at 1 ml/min with the same buffer. In these conditions the LCT-1 was recovered in the flow-through fractions that were pooled, dialysed and lyophilized. These samples were resuspended in 25 mM diethanolamine buffer pH 8.8 and applied to a 8 × 1 cm column of Q-Sepharose (Pharmacia,

© 1993 Rapid Communications of Oxford Ltd

Uppsala, Sweden) and run at 1.1 ml/min in the same buffer. The fractions containing the protein (flow-through) were collected, dialysed against 1 × 100 vol of 20 mM ammonium carbonate buffer pH 8.0 and lyophilized. LCT-5 was purified by applying the yeast medium to a QAE-Zprep 60 disk (Cuno, Meriden, CT, USA) previously equilibrated in 250 mM Tris buffer pH 7.0. After washing with 25 mM Tris pH 7.0, the elution of the protein was performed by applying a gradient of NaCl (0.1–0.5 M) in the same buffer (100 ml). LCT-5 eluted in 0.2 M NaCl and was dialysed and lyophilized as above. To use as negative control in inflammation experiments (see below) a sham protein (SHAM) was prepared using a yeast strain transformed with a plasmid lacking the LCT cDNA and purified as described for LCT-1.

*Western blotting analysis:* Western blots were performed on the proteins separated in 10% polyacrylamide gels and electroblotted to nitrocellulose membranes as described previously.[9] Immunodetection was carried out using specific polyclonal antibodies raised against the amino terminus of the proteins. The amino termini (amino acids 15–31, ENEEQEYVQTVKSSKGG, for LCT-1 and amino acids 1–11, MAQVLRGTVTD, for LCT-5) were synthesized with an Applied Biosystems 430 A peptide synthesizer. The peptides (1 mg/ml in PBS) were cross-linked to 400 μg keyhole limpet haemocyanin (Calbiochem. San Diego, CA, USA) by incubation in 0.5 ml of 20 mM glutaraldehyde at room temperature for 30 min. Antibodies were raised in New Zealand male rabbits by subcutaneous injection of the crosslinked peptides (1 mg in complete Freund's adjuvant). Booster injections of the cross-linked peptides in incomplete Freund's adjuvant were given 7, 14, 24 and 60 days later and rabbits bled 2 weeks after the last injection. Additional Western blots were also performed with polyclonal antibodies raised against the entire proteins (kindly supplied by Dr J. L. Browning, Biogen, Cambridge, MA, USA).

*Animals:* Male Wistar rats (200–250 g body weight) were purchased from Charles River, Calco, Italy and housed for a week before experiments.

*$PGE_2$ release by rat peritoneal macrophages:* Macrophages were collected as described previously.[5] Briefly, rat peritoneal cavities were washed with 20 ml PBS + 5 U/ml heparin (Roche, Basel, Switzerland) and cells plated at $1 \times 10^6$ per well in cluster 24-well plates (Costar, Cambridge, MA, USA), in RPMI 1640 medium (Gibco, Paisley, UK) with 20% foetal calf serum. After 3 h incubation at 37°C in 5% $CO_2$, non-adherent cells were removed by washing with serum-free medium and the adherent macrophages incubated with the proteins for 30 min. After stimulation with 200 μg/ml opsonized zymosan (Sigma, St Louis, MO, USA) for 60 min, in a final volume of 0.5 ml of serum free medium, supernatants were collected and $PGE_2$ concentration assessed by a specific radioimmunoassay (NEN-DuPont, Dreieich, Germany). Cell viability was always greater than 98% as measured by the trypan blue exclusion test.

*Polymorphonuclear leukocyte chemotaxis:* The chemotaxis assay was performed according to Harvarth *et al.*[10] Polymorphonuclear leukocytes (PMN) were collected by rat peritoneal washing (20 ml PBS + 5 U/ml heparin) 4 h after the i.p. injection of 10 ml of 5% thioglycollate (Difco, Detroit, MI, USA). The final suspension contained $1.5 \times 10^6$ cells/ml in the following buffer: 145 mM NaCl, 5 mM KCl, 1 mM $MgSO_4$, 10 mM HEPES, 10 mM glucose, 1 mM $CaCl_2$, pH 7.4. Cell suspension (0.45 ml, $6.75 \times 10^5$ PMN) was incubated with increased concentrations of the proteins (50 μl) at 37°C for 20 min. An aliquot of the suspension was transferred to a 48-well Boyden chemotaxis chamber (Neuro Probe Inc., Bethesda, MD, USA) fitted with 3 μm polycarbonate membranes (Nucleopore, Pleasanton, CA, USA). The migration was induced by 1 μM formyl-methionyl-leucyl-phenylalanine (FMLP, Sigma, St Louis, MO, USA) for 45 min at 37°C in 5% $CO_2$. The migrating cells were stained with Diff-Quik reagent (Merz-Dade AG, Dudingen, Switzerland) and counted with a light microscope. Ten separated fields of each well were counted at 100 ×.

*Carrageenin oedema:* The oedema was induced by the subplantar injection of 0.1 ml of 1% carrageenin (Sigma, St Louis, MO, USA) in 0.9% NaCl into the right hindpaw of rats, as described previously.[5] The proteins were administered i.v. (1 ml/kg) immediately before carrageenin injection (time 0). Control animals received the vehicle (saline). The paw volume was measured in a double blind manner with a water plethysmometer (Basile, Comerio, Italy) at time 0 and every 60 min up to 5 h.

*Zymosan pleurisy:* The pleurisy was induced as described by Perretti *et al.*[11] Briefly, rats were injected intrapleurally with 0.2 ml of 2 mg/ml zymosan in 0.9% NaCl and killed by $CO_2$ inhalation 4 h later. The pleural cavities were washed with 1 ml of PBS + 5 U/ml heparin, the fluids collected and total and differential count of cells performed by using Turk's staining and a Neubauer haematocytometer. The proteins were administered i.v. (1 ml/kg) 30 min before zymosan injection. The doses of the proteins used in the oedema and pleurisy experiments were established on the basis of preliminary experiments. Control animals received the vehicle (saline).

*Statistical analysis:* Statistical significance on original values was assessed by one-tail Student's $t$-test for unpaired samples with $p < 0.05$ regarded as significant.

### Results

*Purity and identification of the proteins:* Figure 1 shows representative SDS-PAGE gels of LCT-5 (lane 1) and LCT-1 (lane 2) after purification. Purity was >90% for LCT-1 and >80% for LCT-5. Figure 1 also shows that the proteins were recognized in Western blots by polyclonal antibodies raised against the entire molecules (lane 3 for LCT-5 and lane 4 for LCT-1) as well as by antibodies raised against the amino termini (lane 5 for LCT-5 and lane 6 for LCT-1).

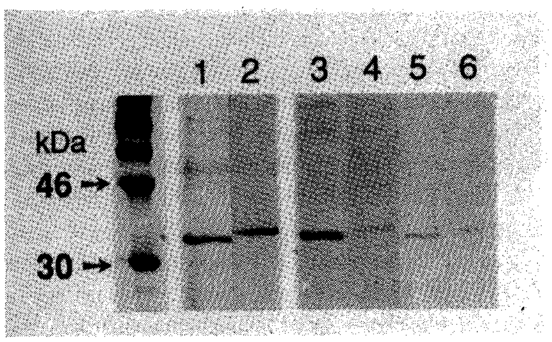

FIG. 1. SDS-PAGE gels of human recombinant LCT-5 (lane 1) and LCT-1 (lane 2) expressed in *S. cerevisiae*. Western blot analysis of LCT-5 (lane 3) and LCT-1 (lane 4) performed with antibodies raised against the whole molecules. Western blot analysis of LCT-5 (lane 5) and LCT-1 (lane 6) performed with antibodies raised against the amino termini.

*Effect of lipocortins on $PGE_2$ release:* LCT-1 reduced in a dose dependent manner the release of $PGE_2$ from activated macrophages with a maximum inhibition of about 70% at 1 ng/ml. LCT-5 showed a weaker effect with a peak inhibition of about 40% at 1 ng/ml. The sham protein preparation had no significant inhibitory effect on $PGE_2$ release (Table 1).

*Effect of lipocortins on PMN chemotaxis:* The chemotaxis of rat PMN was dose dependently reduced by LCT-5 with significant inhibition at 10 and 100 ng/ml (21.8% and 41.7% respectively). LCT-1 caused a 30% inhibition at 1 ng/ml which did not increase at higher concentrations. PMN chemotaxis was not inhibited by the sham protein (Table 2).

*Effect of lipocortins on carrageenin oedema:* The i.v. administration of LCT-1 (1 mg/kg) resulted in a profound and long-lasting reduction of the paw swelling induced by the phlogogen compound. The inhibition was significant at 2, 3 and 4 h after carrageenin injection. In contrast, the same i.v. dose of LCT-5 was completely ineffective in reducing carrageenin oedema. As a matter of fact, the first hour oedema was substantially increased by LCT-5 (Fig. 2).

*Effect of lipocortins on zymosan pleurisy:* The i.v. administration of both LCT-1 and LCT-5 (1 mg/kg) significantly inhibited the migration of PMN and mononuclear (MN) cells induced by the intrapleural injection of zymosan. LCT-5 was more effective

**Table 1.** Effect of LCT-1 and LCT-5 on $PGE_2$ release from zymosan stimulated rat peritoneal macrophages ($n$)

| Protein concentration | % Inhibition | | |
|---|---|---|---|
| | Sham | LCT-1 | LCT-5 |
| 0.01 ng/ml | 7.7 ± 4.5 (3) | 6.4 ± 5.4 (3) | 15.2 ± 5.7 (6) |
| 0.1 ng/ml | 16.4 ± 10.5 (3) | 26.0 ± 9.0* (3) | 11.6 ± 10.0 (6) |
| 1.0 ng/ml | 11.7 ± 7.6 (3) | 64.7 ± 8.8** (6) | 37.3 ± 4.8** (6) |

Results are means ± S.E. of % inhibition *vs.* control values ($PGE_2$ release from macrophages in absence of proteins: 1.8 ± 0.013 ng/ml, $n = 4$). *$p < 0.05$; **$p < 0.01$.

**Table 2.** Effect of LCT-1 and LCT-5 on chemotaxis of rat PMN stimulated with FMLP ($n$)

| Protein concentration | % Inhibition | | |
|---|---|---|---|
| | Sham | LCT-1 | LCT-5 |
| 0.1 ng/ml | ND | 0 (3) | 0 (4) |
| 1 ng/ml | 0 (3) | 29.4 ± 9.0** (9) | 3.0 ± 2.2 (4) |
| 10 ng/ml | 0 (3) | 24.5 ± 8.7 (9) | 21.8 ± 4.5** (13) |
| 100 ng/ml | 0 (3) | 26.8 ± 10.1 (9) | 41.7 ± 4.9** (13) |

Results are means ± S.E. of % inhibition *vs.* control values (migrated cells/well in absence of proteins: 347.9 ± 85.5, $n = 15$). **$p < 0.01$, ND = not done.

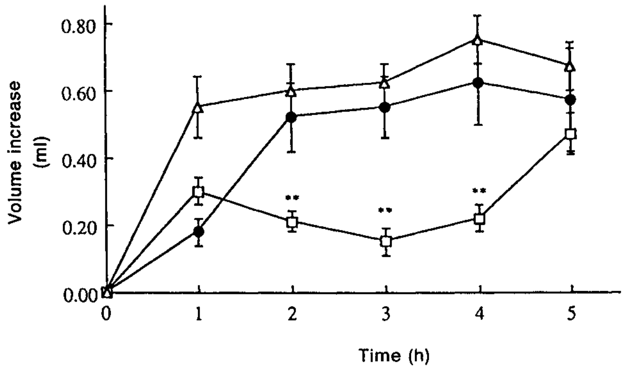

FIG. 2. Effect of LCT-1 and LCT-5 on carrageenin induced paw oedema. Proteins were administered i.v. at time 0. Results are means ± S.E. ($n = 10$ for saline and LCT-5 treated groups, $n = 5$ for LCT-1 treated group). ●, saline; □, LCT-1 1 mg/kg i.v.; △, LCT-5 1 mg/kg i.v. $^{**}p < 0.01$ *vs.* saline treated animals.

than LCT-1 in the inhibition of the migration of either type of cells (PMN −66%, MN −87% for LCT-5; PMN −37%, MN −67% for LCT-1). Leukocyte migration was not significantly modified by i.v. injection of the sham protein (Fig. 3).

### Discussion

The present results indicate that LCT-1 and LCT-5 possess anti-inflammatory activity with some differences. Both proteins inhibited leukocyte migration *in vivo* during zymosan induced pleurisy whereas only LCT-5 caused a dose dependent inhibition of PMN chemotaxis *in vitro*. On the other hand, LCT-5 was less potent than LCT-1 in reducing $PGE_2$ release from activated macrophages and was ineffective in reducing carrageenin induced paw oedema where LCT-1 gave a significant and long-lasting inhibition.

The anti-inflammatory profile of LCT-1 is consistent with inhibition of $PLA_2$ activity. In fact

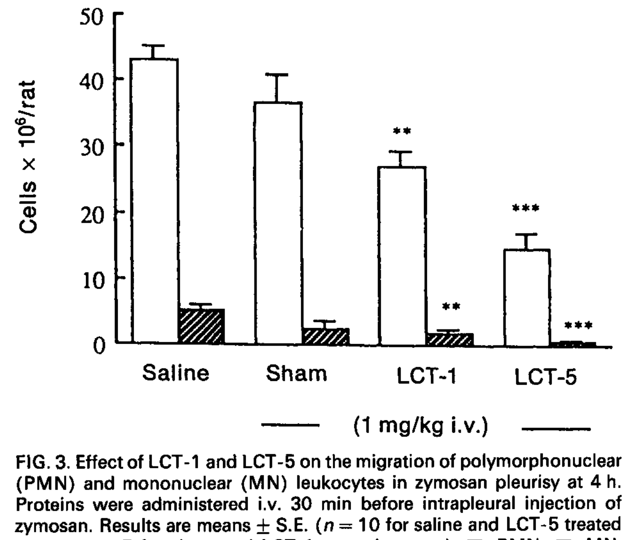

FIG. 3. Effect of LCT-1 and LCT-5 on the migration of polymorphonuclear (PMN) and mononuclear (MN) leukocytes in zymosan pleurisy at 4 h. Proteins were administered i.v. 30 min before intrapleural injection of zymosan. Results are means ± S.E. ($n = 10$ for saline and LCT-5 treated groups, $n = 5$ for sham and LCT-1 treated groups). □, PMN; ▨, MN. $^{**}p < 0.01$; $^{***}p < 0.001$ *vs.* saline treated animals.

the preferential effect on leukocyte migration *in vivo* is likely due to the down-regulation of the synthesis of chemotactic mediators like $LTB_4$ and PAF-acether. LCT-1 is indeed capable, like glucocorticoids, of impairing the formation of all lipid metabolites originating from $PLA_2$ activation.[1] Moreover, LCT-1 and dexamethasone have been shown to reduce PMN migration induced *in vivo* by interleukin-1 (IL-1),[12] which is known to activate $PLA_2$.[13] These data support a regulatory feedback mechanism by LCT-1 and IL-1 on $PLA_2$ activity, as suggested previously.[14] Recently, the presence of saturable binding sites for LCT-1 has been demonstrated on peripheral blood monocytes and neutrophil/s, but not on lymphocytes. The low LCT-1 concentration in plasma results in low receptor occupancy.[15] It is then conceivable that i.v. administered LCT-1 binds to receptors on blood leukocytes reducing the cell capability to migrate into the inflammatory sites. At variance with blood cells, elicited leukocytes present fewer binding sites for LCT-1.[16] This could explain why the inhibitory effect of LCT-1 on chemotaxis *in vitro* reached a plateau at 1 ng/ml and did not increase with concentration. The anti-inflammatory activity of LCT-1 in the carrageenin oedema confirms previous data obtained with a human recombinant protein expressed in *Escherichia coli*[3] whereas other authors did not observe any inhibitory effect with a protein purified from human placenta.[17] This discrepancy is likely due to the different biological activity of the proteins, given the extreme sensitivity of LCT-1 to denaturation and the structural heterogeneity of the various preparations.[18]

The anti-inflammatory profile of LCT-5 suggests that this protein directly affects cell motility in addition to the interference with arachidonic acid metabolism. The carrageenin induced oedema was not inhibited by LCT-5, rather the paw swelling at the first hour was increased by the protein, an effect possibly due to contaminants in the preparation. The lack of inhibition of carrageenin oedema by LCT-5 can be ascribed to the relatively weak effect of the protein on $PGE_2$ release, since prostaglandins are important mediators in this model of inflammation.[19] Since LCT-5 was administered intravenously, it is possible that, in the paw, it did not reach the concentration necessary to inhibit eicosanoid release. This view is supported by the fact that a peptide from LCT-5, injected directly in the paw, was able to inhibit the carrageenin oedema.[5] It is of interest that a 36 kDa protein, related to LCT-5, was able to inhibit PMN migration and eicosanoid formation in a model of murine inflammation. This protein also inhibited PMN chemotaxis *in vitro*.[4] The decreased eicosanoid formation *in vivo* could have been a consequence of the reduced cell migration. The anti-inflammatory effect of LCT-5 could be related to the inhibition of protein kinase C (PKC) activity.[20] It has been suggested that PKC activation in leukocytes leads to cellular responses like lysosomal enzyme release, arachidonate release and superoxide formation.[21] In agreement with this hypothesis it has been shown recently that PKC inhibition results in anti-inflammatory activity.[22] Alternatively or additionally, the inhibitory effect of LCT-5 on cell motility could be due to the property of the protein to tightly bind to negatively charged phospholipids present on activated cell surfaces. It has been shown that LCT-5 binds strongly to membranes of activated macrophages, but not of resting cells.[23] This is supported by the recent observation that peripheral blood leukocytes have very few binding sites for LCT-5 (N. Goulding, personal communication).

Whatever the mechanism of action, which deserves further study, LCT-1 and LCT-5 possess anti-inflammatory activity with some conceivable differences. This observation is very interesting in the light of recent data from the authors' laboratory on the expression of lipocortins brought about by glucocorticoids.[9] The authors have shown that dexamethasone induces the release of LCT-1 and LCT-5, but not of LCT-2, from differentiated U-937 cells. Therefore it can be suggested that glucocorticoids could control the different facets of the inflammatory process through the release of lipocortins endowed with different actions.

### References

1. Flower RJ. Lipocortin and the mechanism of action of the glucocorticoids. *Br J Pharmacol* 1988; **94**: 987–1015.
2. Römisch J, Paques E-P. Annexins: calcium-binding proteins of multi-functional importance? *Med Microbiol Immunol* 1991; **180**: 109–126.
3. Cirino G, Peers SH, Flower RJ, Browning JL, Pepinsky RB. Human recombinant lipocortin 1 has acute local anti-inflammatory properties in the rat paw edema test. *Proc Natl Acad Sci USA* 1989; **86**: 3428–3432.
4. Errasfa M, Russo-Marie F. A purified lipocortin shares the anti-inflammatory effect of glucocorticosteroids *in vivo* in mice. *Br J Pharmacol* 1989; **97**: 1051–1058.
5. Perretti M, Becherucci C, Mugridge KG, Solito E, Silvestri S, Parente L. A novel anti-inflammatory peptide from human lipocortin 5. *Br J Pharmacol* 1991; **103**: 1327–1332.
6. Wallner BP, Mattaliano RJ, Hession C, *et al.* Cloning and expression of human lipocortin, a phospholipase $A_2$ inhibitor with potential anti-inflammatory activity. *Nature* 1986; **320**: 77–81.
7. Pepinsky RB, Tizard R, Mattaliano RJ, *et al.* Five distinct calcium and phospholipid binding proteins share homology with lipocortin 1. *J Biol Chem* 1988; **263**: 10799–10811.
8. Baldari C, Murray JAH, Ghiara P, Cesareni G, Galeotti CL. A novel leader peptide which allows efficient secretion of a fragment of human interleukin 1 beta in *Saccharomyces cerevisiae. EMBO J* 1987; **6**: 229–234.
9. Solito E, Raugei G, Melli M, Parente L. Dexamethasone induces the expression of the mRNA of lipocortin 1 and 2 and the release of lipocortin 1 and 5 in differentiated, but not undifferentiated U-937 cells. *FEBS Lett* 1991; **291**: 238–244.
10. Harvarth L, Falk W, Leonard EJ. Rapid quantitation of neutrophil chemotaxis: use of polyvinylpyrrolidone-free polycarbonate membrane in a multiwell assembly. *J Immunol Meth* 1980; **37**: 39–45.
11. Perretti M, Solito E, Parente L. Evidence that endogenous interleukin-1 is involved in leukocyte migration in acute experimental inflammation in rats and mice. *Agents & Actions* 1992; **35**: 71–78.
12. Perretti M, Flower RJ. Modulation of IL-1-induced neutrophil migration by dexamethasone and lipocortin 1. *J Immunol* 1993; **150**: 992–999.
13. Burch RM, Connor JR, Axelrod J. Interleukin-1 amplifies receptor-mediated activation of phospholipase $A_2$ in 3T3 fibroblasts. *Proc Natl Acad Sci USA* 1988; **85**: 6306–6309.
14. Solito E, Parente L. Modulation of phospholipase $A_2$ activity in human fibroblasts. *Br J Pharmacol* 1989; **96**: 656–660.
15. Goulding NJ, Guyre PM. Regulation of inflammation by lipocortin 1. *Immunol Today* 1992; **13**: 295–297.
16. Perretti M, Flower RJ, Goulding NJ. Murine leukocytes lose lipocortin 1 binding capacity during acute inflammation. *Br J Pharmacol* 1993; in press.
17. Northup JK, Valentine-Braun KA, Johnson LK, Severson DL, Hollenberg MD. Evaluation of the anti-inflammatory and phospholipase-inhibitory activity of calpactin II/lipocortin I. *J Clin Invest* 1988; **82**: 1347–1352.
18. Browning JL, Ward MP, Wallner BP, Pepinsky RB. Studies on structural properties of lipocortin-1 and the regulation of its synthesis by steroids. In: Melli M, Parente L, eds. *Cytokines and Lipocortins in Inflammation and Differentiation.* New York: Wiley-Liss, 1990; 27–45.
19. Di Rosa M, Giroud JP, Willoughby DA. Studies of the mediators of the acute inflammatory response in rats in different sites by carrageenin and turpentine. *J Pathol* 1971; **104**: 15–29.
20. Schlaepfer DD, Jones J, Haigler HT. Inhibition of protein kinase C by annexin V. *Biochemistry* 1992; **31**: 1886–1891.
21. Nishizuka Y. Studies and perspectives of protein kinase C. *Science* 1986; **233**: 305–312.
22. Mulqueen MJ, Bradshaw D, Davis PD, *et al.* Oral, anti-inflammatory activity of a potent, selective, protein kinase C inhibitor. *Agents & Actions* 1992; **37**: 85–89.
23. Römisch J, Schorlemmer U, Fickensher K, Paques E-P, Heimburger N. Anticoagulant properties of placenta protein 4 (annexin V). *Thromb Res* 1990; **60**: 355–366.

**Received 6 January 1993; accepted in revised form 21 January 1993**
